# Supplementary material for: Halogenation of tyrosine perturbs large-scale protein self-organization
Source: Nat Commun. 2022 Aug 17;13:4843. doi: 10.1038/s41467-022-32535-2 (PMC9385671; doi:10.1038/s41467-022-32535-2)
Supplement: Supplementary file 1 — Supplementary Information [file 41467_2022_32535_MOESM1_ESM.pdf]

Supporting Information

Halogenation of Tyrosine perturbs large-scale protein self-organization

Huan Sun[1,2], Haiyang Jia[1,3]*, Olivia Kendall[2,4], Jovan Dragelj[2], Vladimir Kubyshkin[5] Tobias Baumann[2], Maria-Andrea Mroginski[2]*, Petra Schwille[3]* and Nediljko Budisa[2,5]*

1 School of Chemistry and Chemical Engineering, Beijing Institute of Technology, Beijing 100081, P. R. China. 2 Technical University of Berlin, Müller-Breslau-Str. 10, D-10623 Berlin, Germany. 3 Max Planck Institute of Biochemistry, Am Klopferspitz 18, D-82152 Martinsried, Germany. 4 University of Edinburgh, David Brewster Road, King's Buildings, Edinburgh, EH9 3FJ, UK. 5 University of Manitoba, 144 Dysart Rd., R3T 2N2 Winnipeg, Manitoba, Canada.

*Corresponding to: oceanjia0821@gmail.com; andrea.mroginski@tu-berlin.de; schwille@biochem.mpg.de; nediljko.budisa@umanitoba.ca

Supplementary Methods

1. Lipophilicity analysis

The lipophilicity of the amino acid residues was measured against 150 mM buffers as described[1]. The lipophilicity of the amino acid residues was measured against 150 mM buffers: MES buffer (pH 6.0), phosphate buffer (ph 7.0), Tris buffer (pH 8.0) and borate buffer (pH 9.0). All reactions and manipulations were performed at the room temperature: 21-23°C. The starting amino acids and the reagents were of commercial grade. The solvents were of standard grade (“ACS certified”), and they were used without additional purification. NMR spectra were measured at a spectrometer machine operating at 500 MHz 1H frequency at 298 K. First, amino acids were derivatized chemically to yield methyl esters of N-acetylamino acids. An amino acid (1 mmol) was mixed with dichloromethane (10 ml) and N, N-diisopropylethylamine (0.7 ml, 4 equiv.). Trimethylsilylchloride (0.4 ml, 3.2 equiv.) was added, and the mixture was stirred for about 15 min until solution was almost or completely clear. Acetic anhydride was added

in one equivalent (0.1 ml) and the reaction mixture was stirred for about 23 h, and then the reaction vial was left open under a fume hood overnight (20 h) allowing the solvent to evaporate. The residue was dissolved in methanol (2-3 ml), excess of trimethylsilylchloride (0.5 ml) was added, and the mixture was stirred for 60 h. Methanol was removed under reduced pressure, and the target substance was purified by two consecutive short silica gel (about 20 g) columns using ethyl acetate - methanol (40:1 to 20:1) mixture as an eluent. The obtained substance was subjected to distribution experiments as follows. An amino acid derivative (5-10 mg) was mixed with buffer (1 ml) and octan-1-ol (2 ml) in a glass vial, and the vial was vigorously shaken for the next 2-3 hours on a reciprocal laboratory shaker. The vial was centrifuged on a low speed for 1 min to allow full separation of phases. Samples of each phase (0.35 ml) were taken gently by standard 1.00 ml syringes and diluted with DMSO-d6 (0.20 ml) in identical standard 5 mm NMR tubes. 1H NMR spectra were measured in one scan and multiscan in datasets with identical settings for samples of both phases. The datasets were copied and applied for measurements without readjustment of acquisition or processing parameters (except zero-order phase) and without readjustment of the receiver gain. One-scan and multi-scan (8*n) measurement were performed on the samples. A single 90-degree pulse experiment with no dummy scans was used for single scan experiments, and a 30-degree pulse experiment with 8 dummy scans was used for collection of multiscan spectra. The spectra were processed in a conventional manner using Fourier transform with 1-2 Hz line broadening and a 5-degree polynomial baseline correction. The comparison of the relative analyte concentration in the phases was made by comparison of the absolute integral values and/or by overlaying resonances from two spectra on each other, whichever was more accurate. The intensities of the resonances were compared by overlay and integration of the spectra. A ratio between resonance intensity in octan-1-ol and buffer phase was considered a distribution coefficient. For each pH/substance combination the distribution experiments were performed in triplicate. DpH values were read out for several separate resonances for each sample. Averaging these values produced the final DpH value and

the standard deviation for a compound/pH combination. N-Acetyl-tyrosine methyl ester and N-acetyl-3-iodo-tyrosine methyl ester were reported earlier, and other compounds were synthesized according to the protocol. The final distribution coefficients logD are provided in Supplementary Table 7. A dedicated article on the used method and reference datasets is available in reference 1.

N-Acetyl-3-chloro-tyrosine methyl ester: ^{1}H NMR (DMSO-d_6, 500 MHz), δ: 9.98 (s, 1H, OH), 8.29 (d, *J* = 7.7 Hz, 1H, NH), 7.18 (d, *J* = 1.6 Hz, 1H, ArH), 6.97 (dd, *J* = 8.3 1.6 Hz, 1H, ArH), 6.86 (d, *J* = 8.3 Hz, 1H, ArH), 4.38 (ddd, *J* = 9.2, 7.8, 5.6 Hz, 1H, α-CH), 3.60 (s, 3H, CH_3O), 2.90 (dd, *J* = 13.8, 5.5 Hz, 1H, $C\underline{H}H$), 2.75 (dd, *J* = 13.8, 9.3 Hz, 1H, $CH\underline{H}$), 1.80 (s, 3H, CH3CO). 13C{1H} NMR (DMSO-d_6, 126 MHz), δ: 172.6 (ester CO), 169.8 (amide CO), 152.1 (C), 130.7 (CH), 129.4 (C), 129.0 (CH), 119.7 (C), 116.9 (CH), 54.1 (α-CH), 52.2 (CH_3O), 36.0 (CH_2), 22.7 (CH_3).

N-Acetyl-3-bromo-tyrosine methyl ester: ^{1}H NMR (DMSO-d_6, 500 MHz), δ: 10.06 (s, 1H, OH), 8.29 (d, *J* = 7.8 Hz, 1H, NH), 7.32 (d, *J* = 2.1 Hz, 1H, ArH), 7.01 (dd, *J* = 8.3, 2.1 Hz, 1H, ArH), 6.85 (d, *J* = 8.3 Hz, 1H, ArH), 4.38 (ddd, *J* = 9.2, 7.8, 5.6 Hz, 1H, α-CH), 3.60 (s, 3H, CH_3O), 2.90 (dd, *J* = 13.9, 5.6 Hz, 1H, $C\underline{H}H$), 2.75 (dd, *J* = 13.8, 9.3 Hz, 1H, $CH\underline{H}$), 1.80 (s, 3H, CH_3CO). $^{13}C\{^{1}H\}$ NMR (DMSO-d_6, 126 MHz), δ: 172.6 (ester CO), 169.8 (amide CO), 153.1 (C), 133.7 (CH), 129.8 (C), 129.7 (CH), 116.6 (CH), 109.3 (C), 54.1 (α-CH), 52.2 (CH_3O), 35.9 (CH_2), 22.7 (CH_3).

N-Acetyl-3-iodo-tyrosine methyl ester: ^{1}H NMR (DMSO-d_6, 500 MHz), δ: 10.14 (s, 1H, OH), 8.28 (d, *J* = 7.7 Hz, 1H, NH), 7.51 (d, *J* = 2.1 Hz, 1H, ArH), 7.03 (dd, *J* = 8.2, 2.1 Hz, 1H, ArH), 6.78 (d, *J* = 8.2 Hz, 1H, ArH), 4.36 (ddd, *J* = 9.0, 8.0, 5.6 Hz, 1H, α-CH), 3.60 (s, 3H, CH_3O), 2.88 (dd, *J* = 13.9, 5.6 Hz, 1H, $C\underline{H}H$), 2.73 (dd, *J* = 13.8, 9.2 Hz, 1H, $CH\underline{H}$), 1.80 (s, 3H, CH_3CO). $^{13}C\{^{1}H\}$ NMR (DMSO-d_6, 126 MHz), δ: 172.6 (ester CO), 169.8 (amide CO), 155.7 (C), 139.6 (CH), 130.6 (CH), 130.2 (C), 115.2 (CH), 84.7 (C), 54.2 (α-CH), 52.2 (CH_3O), 35.8 (CH_2), 22.7 (CH_3).

N-Acetyl-3,5-dichloro-tyrosine methyl ester: ^{1}H NMR (DMSO-d_6, 500 MHz), δ: 9.96

(s, 1H, OH), 8.31 (d, J = 7.9 Hz, 1H, NH), 7.23 (s, 2H, ArH), 4.42 (ddd, J = 9.5, 7.9, 5.4 Hz, 1H, α-CH), 3.61 (s, 3H, CH_3O), 2.94 (dd, J = 13.9, 5.4 Hz, 1H, C$\underline{H}$H), 2.76 (dd, J = 13.8, 9.7 Hz, 1H, CH$\underline{H}$), 1.80 (s, 3H, CH_3CO). $^{13}C\{^1H\}$ NMR (DMSO-d_6, 126 MHz), δ: 172.4 (ester CO), 169.8 (amide CO), 148.0 (C), 130.8 (C), 129.6 (CH), 122.3 (C), 53.7 (α-CH), 52.3 (CH_3O), 35.6 (CH_2), 22.7 (CH_3).

N-Acetyl-3,5-dibromo-tyrosine methyl ester: ^1H NMR (DMSO-d_6, 500 MHz), δ: 9.76 (s, 1H, OH), 8.32 (d, J = 8.0 Hz, 1H, NH), 7.40 (s, 2H, ArH), 4.42 (ddd, J = 9.6, 8.0, 5.6 Hz, 1H, α-CH), 3.61 (s, 3H, CH_3O), 2.94 (dd, J = 13.8, 5.4 Hz, 1H, C$\underline{H}$H), 2.76 (dd, J = 13.8, 9.6 Hz, 1H, CH$\underline{H}$), 1.80 (s, 3H, CH_3CO). $^{13}C\{^1H\}$NMR (DMSO-d_6, 126 MHz), δ: 172.4 (ester CO), 169.8 (amide CO), 149.7 (C), 133.3 (CH), 132.2 (C), 112.1 (C), 53.8 (α-CH), 52.3 (CH_3O), 35.4 (CH_2), 22.7 (CH_3).

N-Acetyl-3,5-diiodo-tyrosine methyl ester: ^1H NMR (DMSO-d_6, 500 MHz), δ: 9.39 (s, 1H, OH), 8.30 (d, J = 8.0 Hz, 1H, NH), 7.59 (s, 2H, ArH), 4.37 (ddd, J = 9.4, 8.0, 5.6 Hz, 1H, α-CH), 3.60 (s, 3H, CH_3O), 2.89 (dd, J = 13.8, 5.4 Hz, 1H, C$\underline{H}$H), 2.72 (dd, J = 13.8, 9.5 Hz, 1H, CH$\underline{H}$), 1.80 (s, 3H, CH_3CO). $^{13}C\{^1H\}$ NMR (DMSO-d_6, 126 MHz), δ: 172.4 (ester CO), 169.7 (amide CO), 154.4 (C), 140.1 (CH), 133.9 (C), 87.3 (C), 53.9 (α-CH), 52.3 (CH_3O), 35.0 (CH_2), 22.7 (CH_3).

NMR spectra for *N*-acetyl-3-halo-tyrosine methyl esters:

^{1}H NMR (DMSO-d$_6$, 500 MHz):

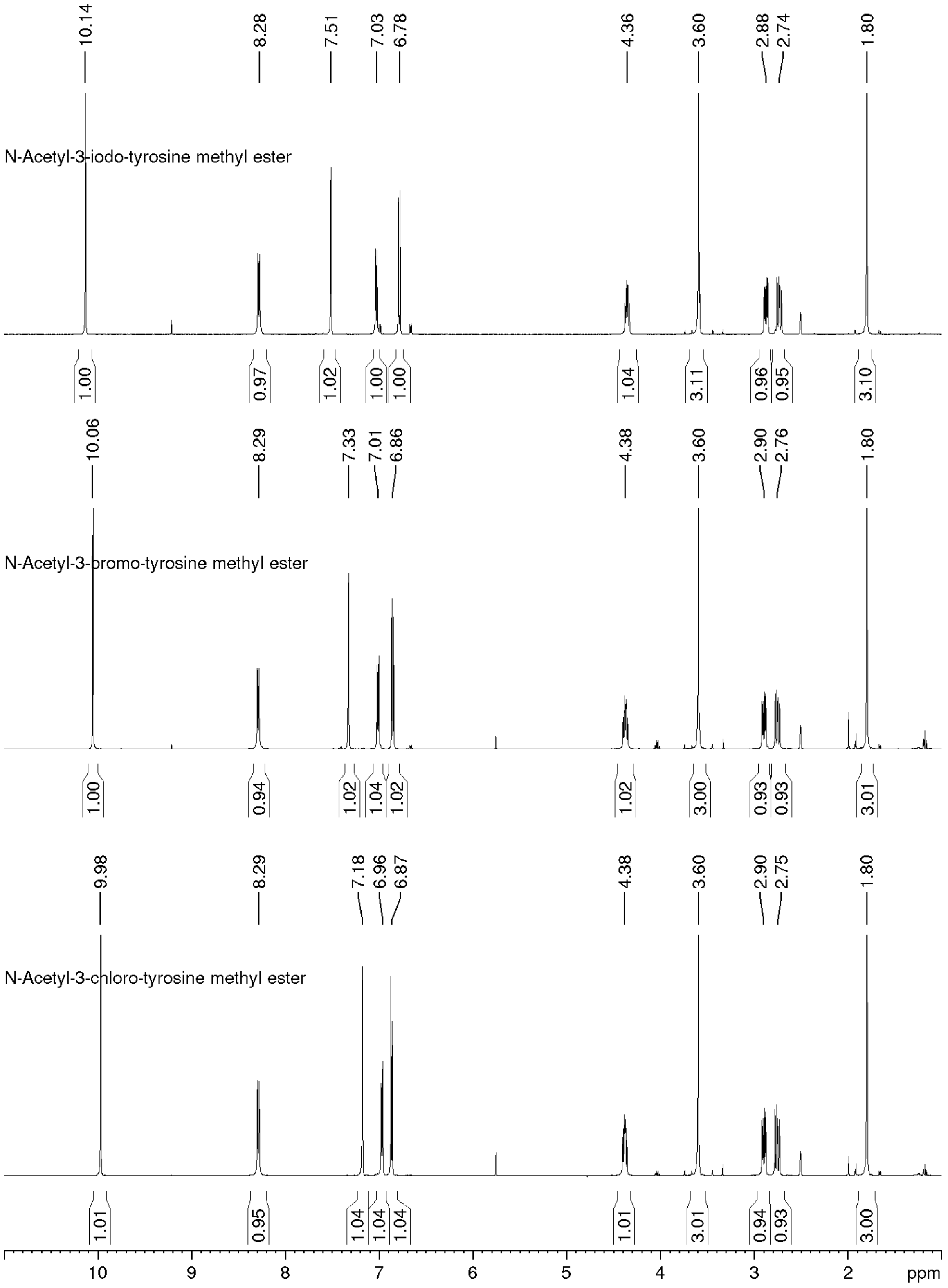

$^{13}C\{^{1}H\}$ NMR (DMSO-d_6, 126 MHz):

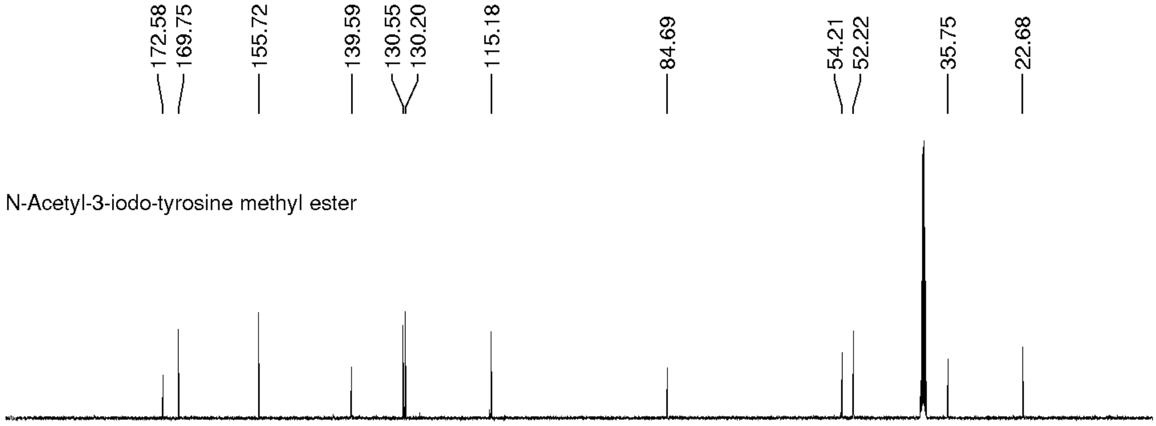

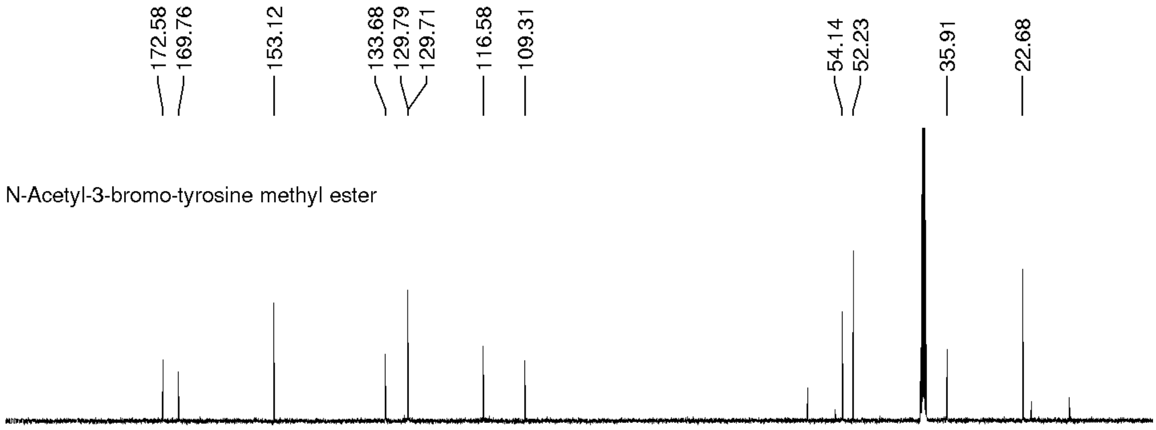

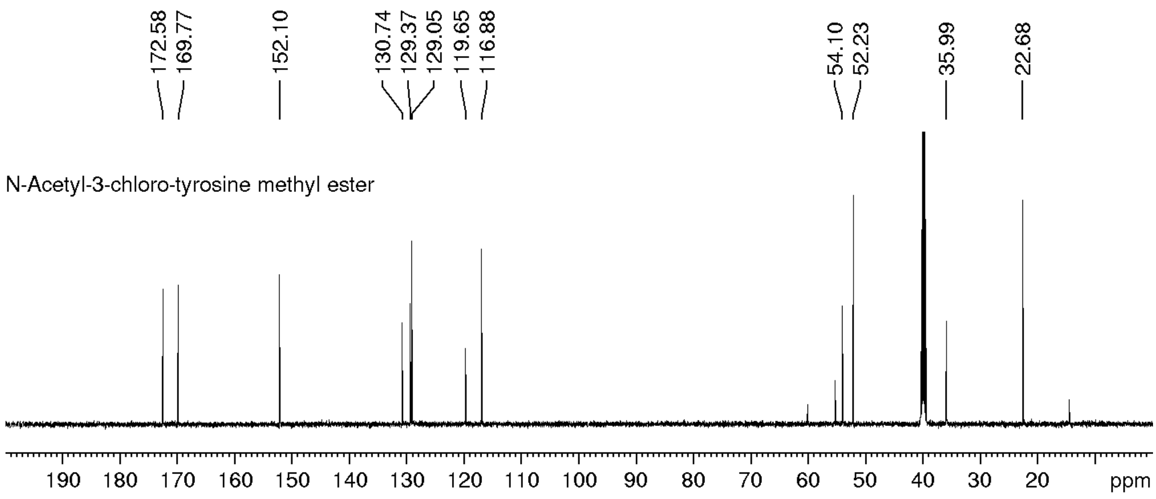

NMR spectra for *N*-acetyl-3,5-dihalo-tyrosine methyl esters:

^{1}H NMR (DMSO-d$_6$, 500 MHz):

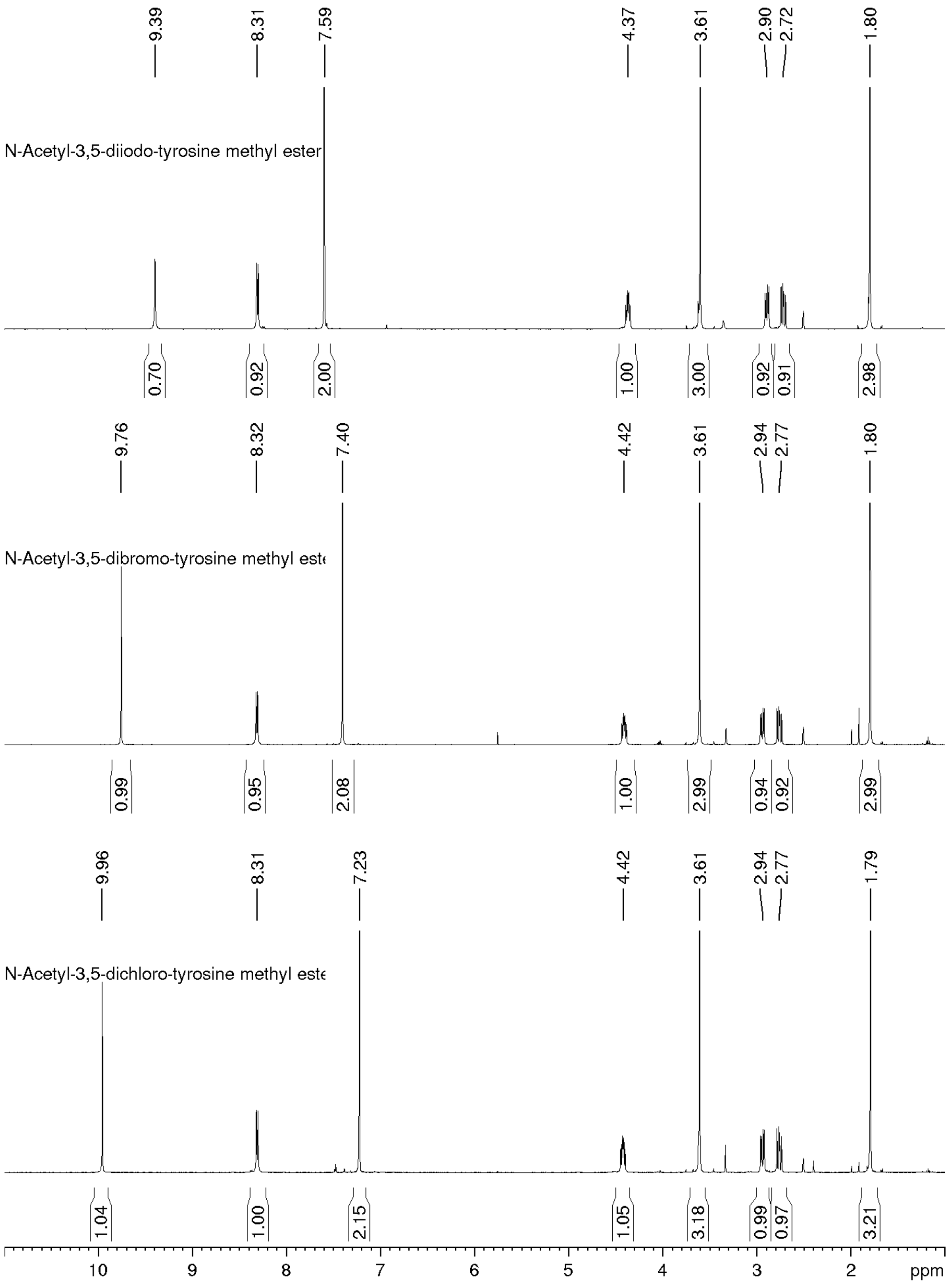

$^{13}C\{^{1}H\}$ NMR (DMSO-d$_6$, 126 MHz):

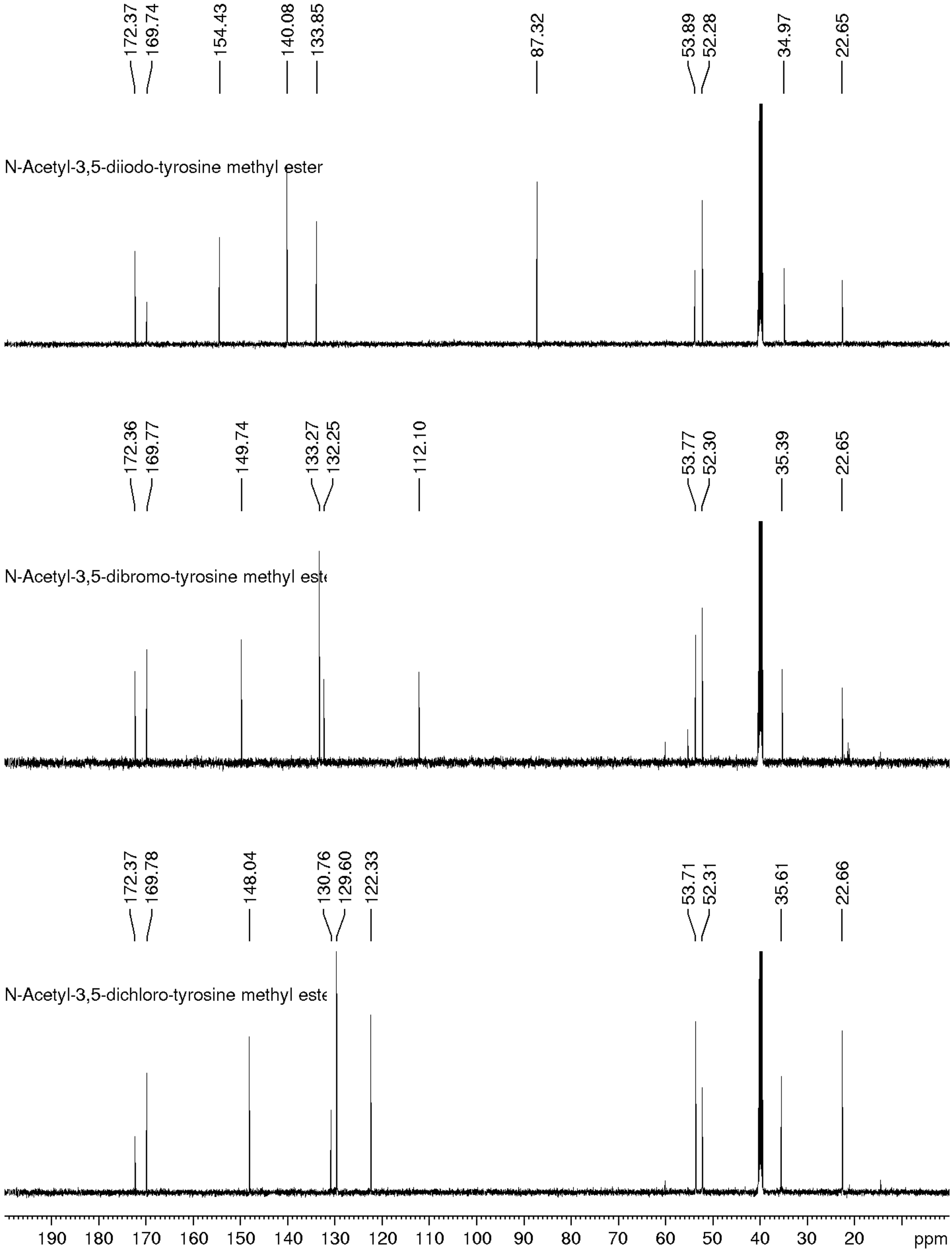

2. FtsZ structure simulation

Homology modelling

A homology model was created using the I-TASSER suite[2](templates from *M. Jannaschii,* PDB code: 1W5A)[3]. Secondary structure refinements were performed using Modeller software[4].

Wild-type structure preparation

The initial homology model was first relaxed by a 10 ns MD simulation run to ensure a thermally equilibrated molecule was obtained. Titrations were performed using Karlsberg^{2+} on CUDA platforms, utilising a cavity algorithm with constant 0.7[5,6]. GTP and bound Mg^{2+} positions were taken by aligning 1W5A to the homology model with the MultiSeq tool in VMD[4,7]. A preliminary minimisation procedure of 2000 steps in a protein dielectric constant $\varepsilon = 4$ was used to optimise side chains of residues within 8 Å of GTP; all other atoms were constrained. This model was used as a monomer reference structure in future calculations.

Mutant structure preparation

Crystal structures containing the mutated residues were found as the basis structures for the modified tyrosine (PDB codes: 4NX2 for Cl and 3GFD for I mutant respectively)[8,9]. A methyl group replaced CA and the backbone for the purposes of charge calculation. Geometry optimisation of the hydrogen atoms was performed whilst heavy atoms were constrained, and the electrostatic potential (ESP) was calculated using the Gaussian 16 program[10]. Different basis sets were used according to the number of orbitals required for each halogen: 6-31G* for chlorine[11], 3-21G for iodine[12]. Partial charges were calculated using RESP tools in the Amber antechamber[13]. The optimised phenyl ring was aligned with tyrosine ring of the reference structure and merged with the backbone. Charges for the backbone were taken from the tyrosine residue in the Amber force field. The charge of the methyl substituent of the ring was redistributed in the residue to give an overall neutral charge; final charges are shown in Supplementary Table 5.

Bond, angle, dihedral and improper dihedral parameters were calculated based on the methyl ring with optimized hydrogens, merged with the backbone of the reference structure; the backbone was necessary to ensure all dihedrals could be calculated and to enable Amber to recognize the main chain atoms that connect the residue to the rest of the protein. This was done using the antechamber package of Amber[13]. All necessary parameters were taken from the standard parm10 database.

Dimer structure preparation

The 2004 *M. Jannaschii* dimer structure (1W5A) was used as a template, on which the monomer input structures were aligned using the MultiSeq plugin of VMD[14]. As a result of this, the N-terminal tail of the upper monomer pointed inwards and clashed with the dimer interface, which would lead to the calculation of large repulsive forces for the tail and a system crash during the minimization procedure. Therefore, the monomer PDB was aligned to the first 12 residues of each chain in the template structure. The coordinates were then saved and merged with the monomer PDB from the backbone nitrogen atom at the 11 position. The preliminary dimer structure was minimized using a dielectric constant of 4, in order to relax the protein structure. This model was used as a dimer reference structure in later analysis.

Molecular dynamics simulation

In this study, all molecular dynamics (MD) modelling was performed using the Amber software suite[7], using the recommended ff14SB, TIP3P and gaff2 force fields for protein, water, and generalised molecules respectively[15] and in-house developed GTP parameters[16]. In each simulation, the protein was solvated in a TIP3P[17] periodic box, measuring 12 Å from the edges of the protein. The system was neutralized with K^+ ions, which were distributed based on a Coulombic potential grid. No further ions were added, apart from the Mg^{2+} ions associated with the active site. The energy of the system was minimized for 12 000 steps, the first 10 000 steps using the steepest descent method. The system was then heated from 0 K to 300 K over the course of 500 ps using

an NVT ensemble, during which backbone atoms were constrained. Langevin dynamics were used to control the temperature with a collision frequency of 2.0 ps^{-1} [18]. The system was equilibrated for 200 ps without constraints on backbone atoms preceding production. A 2 fs integration time-step was used for the simulation and the SHAKE algorithm[19] was employed to constrain hydrogen bonds during the simulation. Non-bonded terms were cut off at 12 Å; particle mesh Ewald (PME) was used to treat long-range electrostatics[20]. Simulations were performed on a CUDA- accelerated platform with 12 cores at 3.0 GHz and 128 GB RAM.

Supplementary Figures

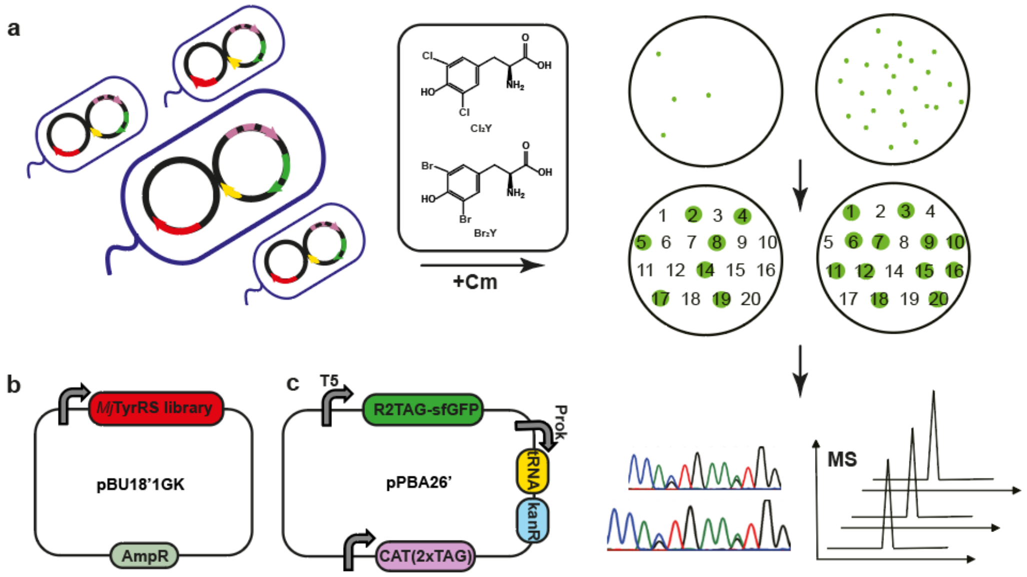

Supplementary Figure 1. One round positive selection method to identify specific aaRS variants for orthogonal activating a halogenated Tyr analogue**.** a.) *E. coli* strain DH10b co-transformed with *Mj*TyrRS gene library plasmid (pBU18'1GK_*Mj*TyrRS_library) and selection plasmid containing chloramphenicol acetyltransferase (*cat*) and small ubiquitin-like modifier tagged sfGFP bearing stop codon is grown in the presence of a specific HY and proper concentration of chloramphenicol (Cm). The cells containing the specific *Mj*TyrRS variants could only survive and have green fluorescence in the presence of HYs in the medium with Cm. The promising clones are sequenced and MS is used to confirm the fidelity of HYs incorporation by screened *Mj*TyrRSs. b-c.) *Mj*TyrRS gene library plasmid (pBU18'1GK_MjTyrRS_library) and the dual reporter selection plasmid (pPAB26_cat (Q98TAG, D181TAG) *Mj*tRNATyCUA_his_SUMO-sfGFPR2TAG-strep). pPAB26 has a p15A ori, a Kan resistance for plasmid propagation, and one copy of the suppressor *Mj*tRNATyr CUA, controlled by proK promoter. It contains a CAT encoding gene bearing amber codons at position Gln98 and Asp181 and SUMO fused sfGFP bearing TAG at Arg-2 position driven by T5 promoter.

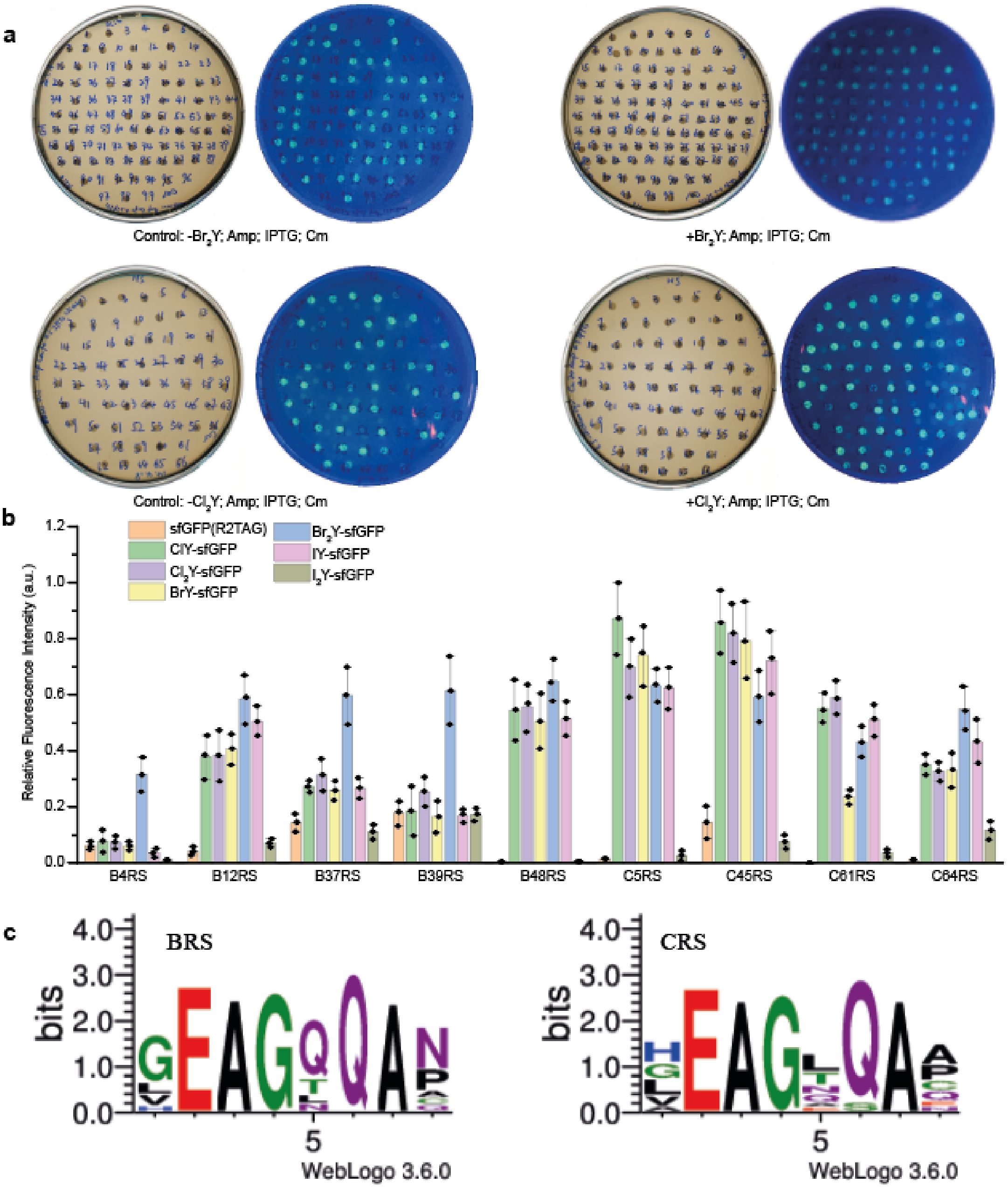

Supplementary Figure 2. Selective incorporation of halogenated tyrosine analogues by one-round positive selection. a.) Growth assay of *E. coli* (DH10b) cell for evaluating the one-round positive selected *Mj*TyrRS towards Br_2Y (BRS) and Cl_2Y (CRS). Re-streak of 100 colonies containing dual reporter selection plasmid and the potential BRSs encoding gene plasmid possible on new minimum medium[21] agar plates supplied with 100μg/mL Amp, 50μg/mL Kan, 0.5 mM IPTG and 70μg/mL Cm in presence or absence of 1mM Br_2Y or Cl_2Y. The represent fluorescence photos were taken under UV light. b.) Validation and characterization of HYs incorporation into SUMO-sfGFP

bearing an amber codon at Arg-2 by 9 unique synthetase mutants selected in this study (B4RS, B12RS, B37RS, B39RS, B48RS, C5RS, C45RS, C61RS, and C64RS). sfGFP fluorescence assay of DH10b containing selected *Mj*TyrRS variants in the absence of HY or presence of ClY, Cl_2Y, BrY, Br_2Y, IY and I_2Y, respectively. Fluorescence values (excitation: 481 nm, emission: 511nm) are normalized to the lowest fluorescence value in DH10b and to the OD_{600} of the bacterial culture. The data shown was an average of triplicate measurements (Mean ± s.d.). (c) The amino acid sequences of *Mj*TyrRS variants for recognizing Br_2Y and Cl_2Y are represented by WebLogo[22]. The amino acid type and randomized positions are shown on the x-axis. The overall height of the amino acid stacks plotted on the y-axis, indicates the sequence conservation at a given position, while the height of individual symbols within a stack indicates the relative frequency of an amino acid at that position. The colors of the amino acids correspond to their chemical properties; polar amino acids are shown in green, hydrophobic amino acids are shown in black, acidic amino acids are shown in red, basic amino acids are shown in blue, neutral amino acids are shown in purple. Source data of b. are provided as a Source Data file.

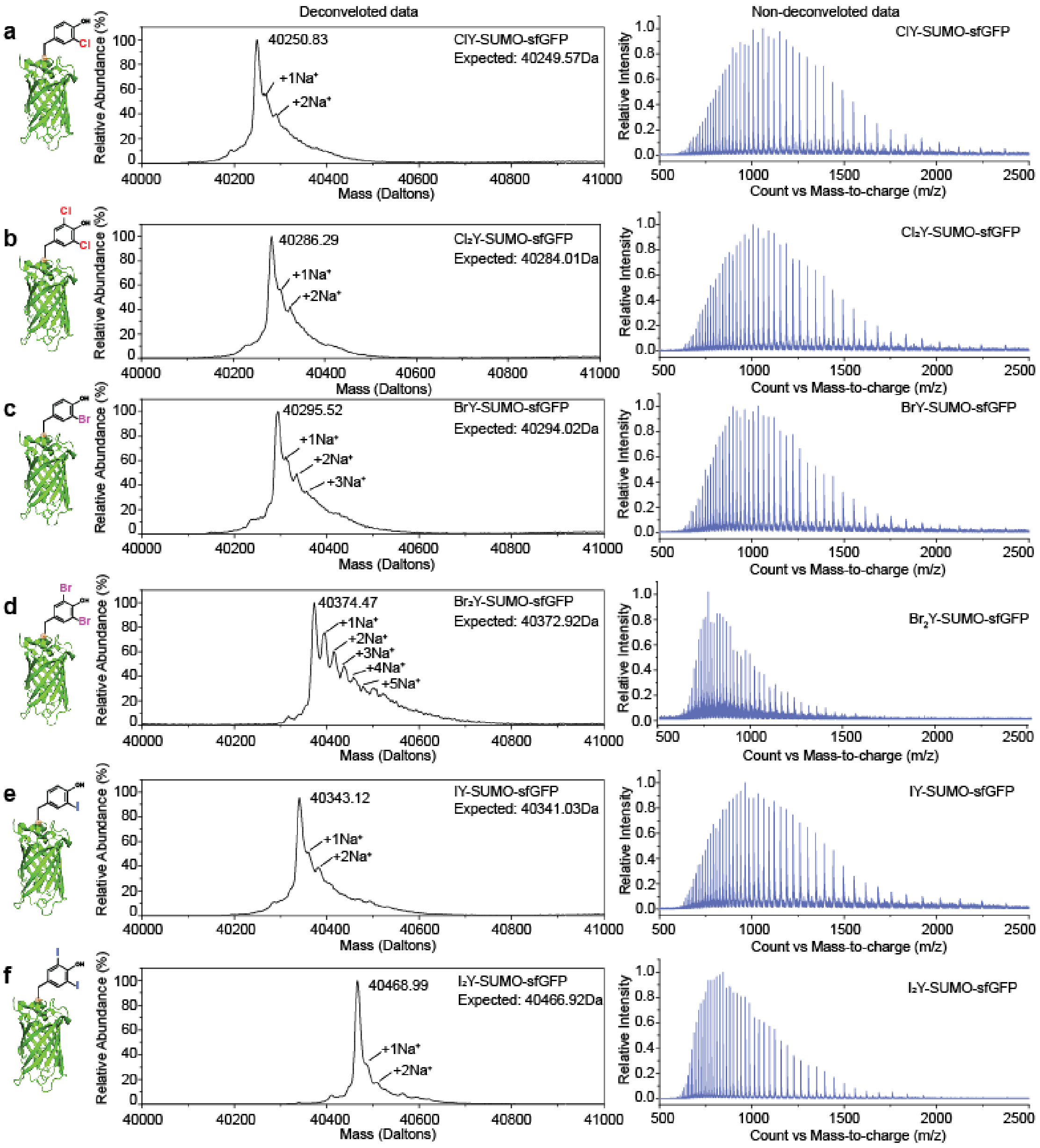

Supplementary Figure 3. The deconvoluted and non-deconvoluted ESI-MS spectra of SUMO-sfGFPR2TAG incorporated with HYs. The observed and expected molecular masses are as follows: a.) ClY-SUMO-sfGFP: observed:40250.83Da, expected: 40249.57Da. b.) Cl_2Y-SUMO-sfGFP: observed:40286.29Da, expected: 40284.01Da. c.) BrY-SUMO-sfGFP: observed: 40295.52Da, expected: 40294.02Da. d.) Br_2Y-SUMO-sfGFP: observed: 40374.47Da, expected: 40372.92Da. e.) IY-SUMO-sfGFP: observed: 40343.12Da, expected: 40341.03Da. f.) I_2Y-SUMO-sfGFP: observed: 40468.99Da, expected: 40466.92Da. Sodium adduct species are indicated in the figure. Source data of a-f. are provided as a Source Data file.

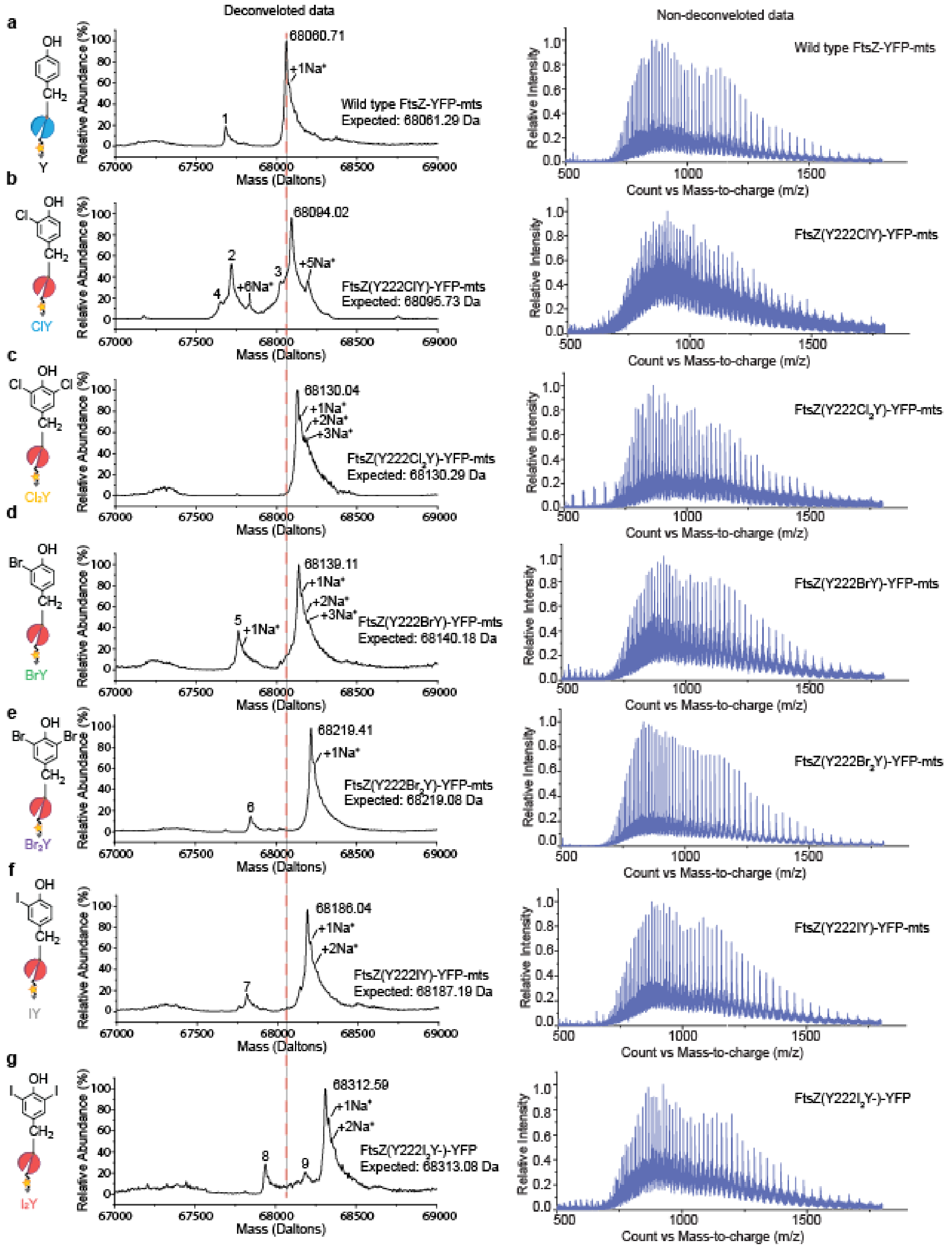

Supplementary Figure 4. The deconvoluted and non-deconvoluted ESI-MS spectra of FtsZ-YFP-mts incorporated with HYs. The observed and expected molecular masses are as follows: a.) wild type FtsZ-YFP-mts (Y): main peak (observed: 68060.71 Da, expected: 68061.29 Da). b.) FtsZ(Y222ClY)-YFP-mts: main peak (observed: 68094.02 Da, expected: 68095.73 Da); c.) FtsZ(Y222Cl_2Y)-YFP-mts: main peak: observed:

68130.04 Da, expected: 68130.29 Da. d.) FtsZ(Y222BrY)-YFP-mts: main peak (observed: 68139.11 Da, expected: 68140.18 Da). e.) FtsZ(Y222 Br_2Y)-YFP-mts: main peak (observed: 68219.41 Da, expected: 68219.08 Da). f.) FtsZ(Y222IY)-YFP-mts: main peak (observed: 68186.04 Da, expected: 68187.19 Da). g.) FtsZ(Y222I_2Y)-YFP-mts: main peak (observed: 68312.59 Da, expected: 68313.08 Da). All the satellite peaks are listed below: Peak1 (67685.53 Da), Peak2 (67719.94 Da), Peak5 (67765.03 Da), Peak6 (67844.63 Da), Peak7 (67808.00 Da) and Peak8 (67937.56 Da) represent the proteins that are around 375 Da smaller compared to the main peak, which probably resulted from the loss the of C-terminal α-helix of FtsZ-YFP-mts during ESI-MS measurement. The mts from MinD is a positively charged domain that is unstable under the acidic conditions applied in the ESI-MS measurement protocol due to a low ionic strength[23]. It has been reported that the N-terminal or C-terminal α-helices can be prone to a loss under low ionic strength[24]. Peak3 (observed: 68027.10 Da, expected: 68,027.52 Da) indicates FtsZ(Y222ClY)-YFP-mts without N-terminal Met but with 3 Na^+ adduct. Peak4 (observed: 67652.90 Da, expected: 67,652.52 Da) represents FtsZ(Y222ClY)-YFP-mts without N-terminal Met and C-terminal mts (during ESI-MS measurement) but with 3 Na^+ adduct. Peak 9 (observed: 68186.01 Da, expected: 68186.69 Da) is deiodination of FtsZ(Y222 I_2Y)-YFP-mts caused by formic acid during MS measurement[25]. Source data of a-g. are provided as a Source Data file.

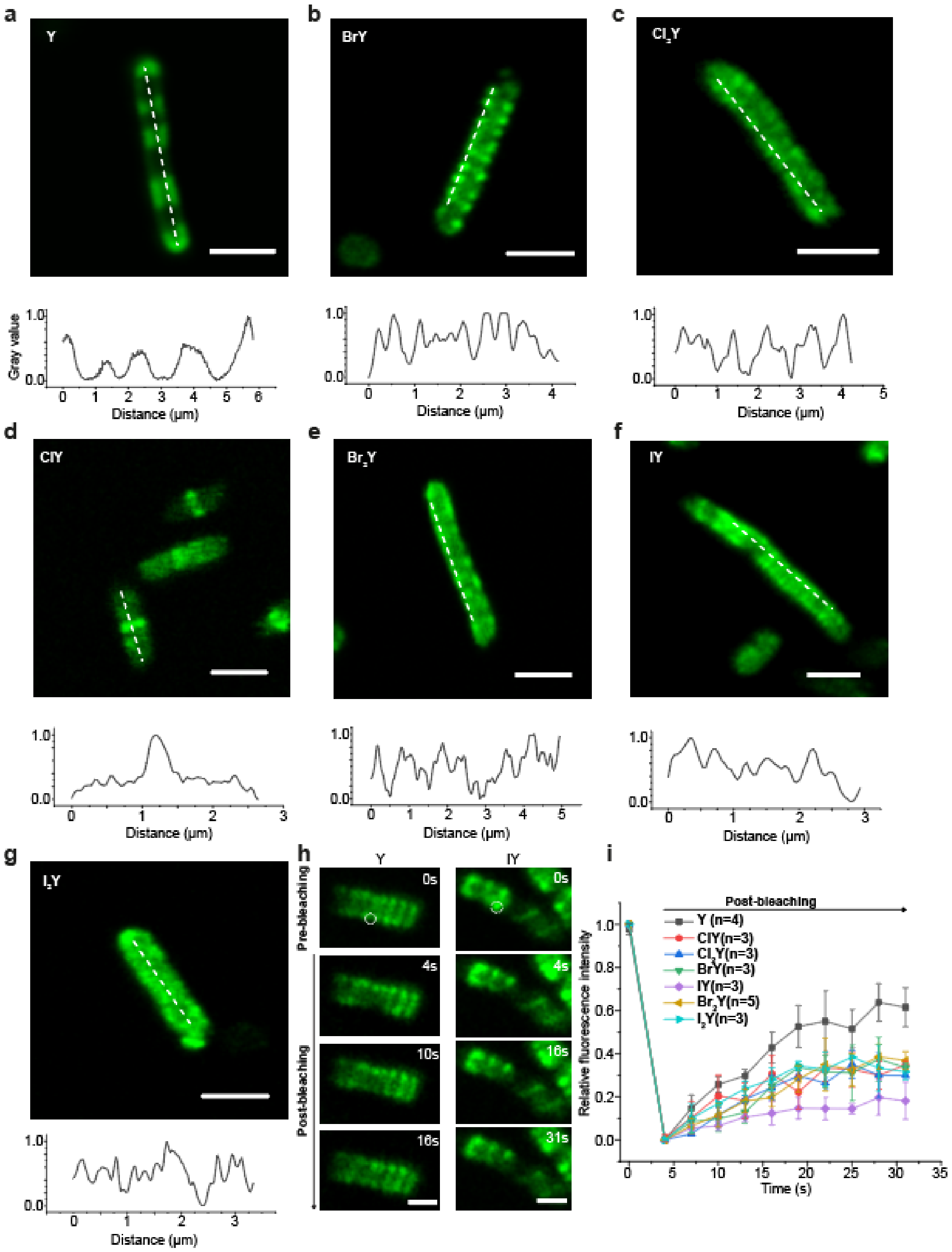

Supplementary Figure 5. Self-assembly of halogenated FtsZ in live *E. coli* cells. Localization and patterns of a.) Wild type FtsZ-YFP-mts (Y), b.) BrY, c.) Cl_2Y, d.) ClY, e.) Br_2Y, f.) IY, and g.) I_2Y incorporated FtsZ-YFP-mts in living *E. coli* cells. Scale bar, 2μm. The white lines indicate the position for the line plot. h-i) FRAP analysis of dynamics of FtsZ in *E. coli*. h.) Representative snapshots of *E. coli* cell overexpressed with wild type FtsZ-YFP-mts and FtsZ(Y222IY)-YFP-mts after photo-bleaching. Scale

bar: 1μm. i.) Fluorescence recovery curves of wild type FtsZ-YFP-mts and halogenated FtsZ(Y222X)-YFP-mts (X = ClY, BrY, Br_2Y, I_2Y, Cl_2Y, and IY) after photo-bleaching. Data points in i. are shown as means with standard deviations. The replicate numbers are indicated in the figure. Source data of a-g. and i. are provided as a Source Data file.

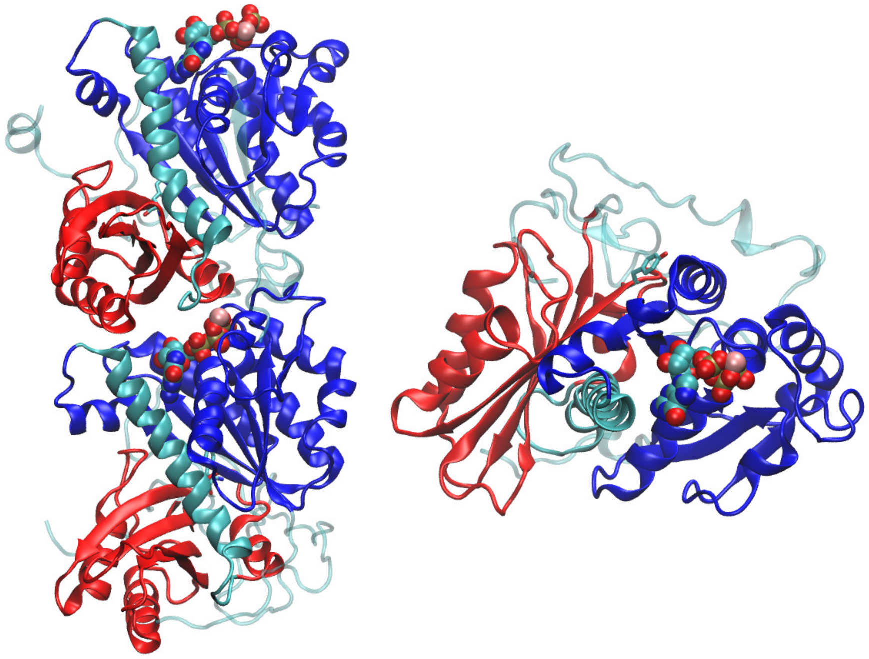

Supplementary Figure 6. Dimer (left) and monomer (right) of FtsZ structures. The monomer is rotated 90 degrees, looking down the main axis of the dimer and looking into the GTP binding pocket. The main protein body is shown in cartoon representation, GTP is displayed in Van der Waals representation and tyrosine 222 is displayed in licorice representation. The NTD is colored blue, CTD is red and the central H7 helix is shown in cyan. The C-terminal tail is displayed in transparent cyan.

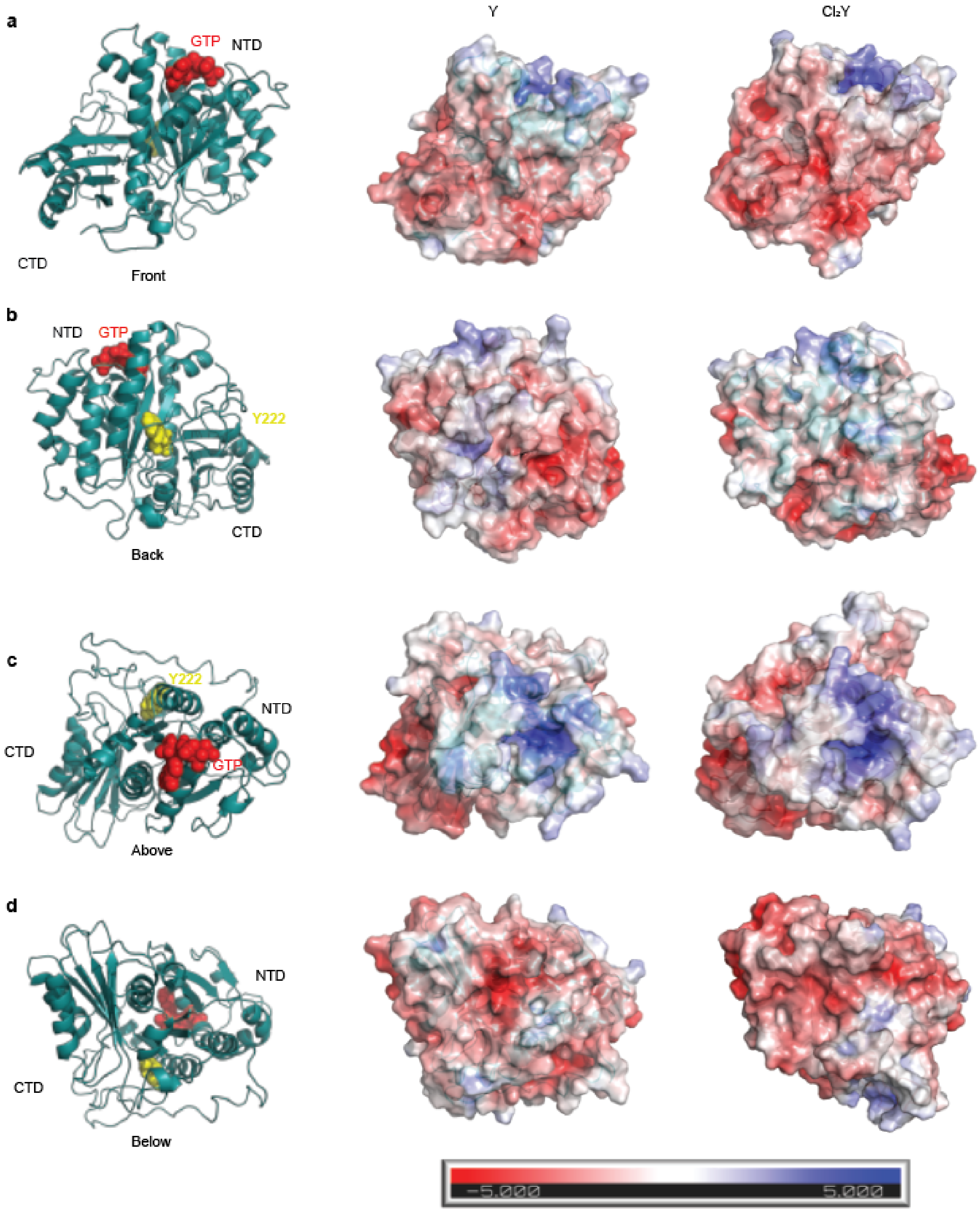

Supplementary Figure 7. EPS surface of monomer conformations at 500 ns from. a.) "front", facing H7 helix, b.) "back", 180° rotation around y axis and facing CTT, c.) "above", 90° rotation around x axis and facing into GTP binding pocket and NTD interface in dimer, d.) "below", 180° around x axis and facing T7 loop and CTD interface in dimer (depicted for Wild type FtsZ in cartoon representation). Calculated using Pymol APBS tools using the dielectric constants ε = 80 for protein and ε = 4 for water. Surface shown for range ±5 eV.

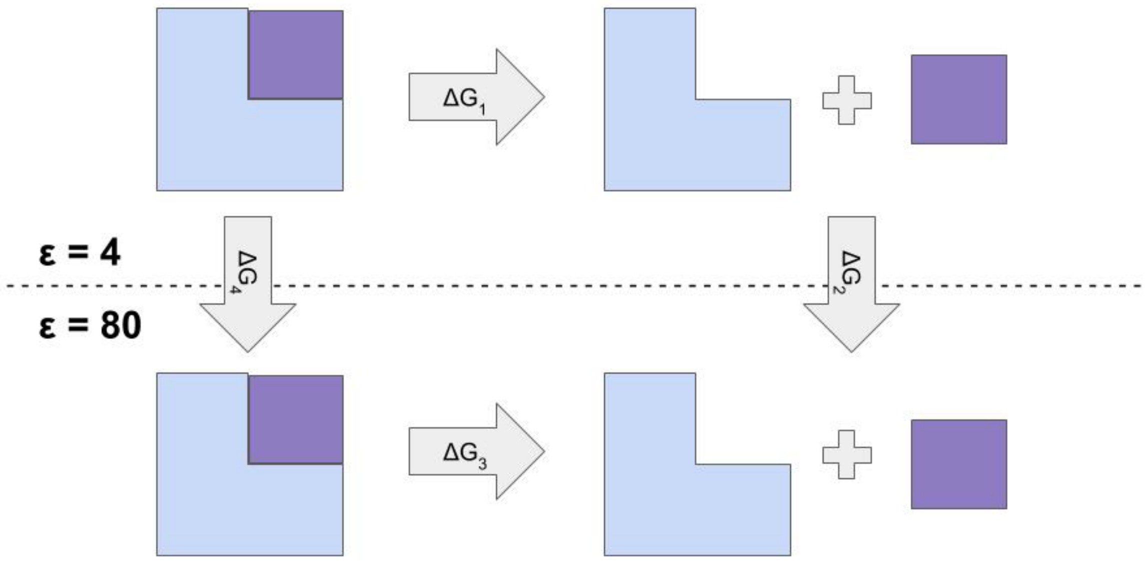

Supplementary Figure 8. Depiction of solvation binding energy scheme. Solvent dielectric constant, ε, used for each system is shown in bold on left hand side, solute (protein/GTP moiety) dielectric constant ε = 4 for all calculations. Evaluation of the solvation binding energies shown in Fig. 4c were calculated according to this scheme. ΔG_1 signifies the coulombic energy of complex formation from its individual components, where a homogeneous dielectric constant (ε = 4) is used to represent the solvent and the solute. ΔG_2 and ΔG_4 represent the free energy difference gained when the solute is moved to a heterogeneous dielectric environment (solvent, ε = 80; solute, ε = 4) from a homogeneous dielectric environment (ε = 4). Here we do not assume that FtsZ is in the membrane, but rather in solution and the value of 4 refers to the protein dielectric constant. The protein dielectric value of 4 was chosen in order to allow proper treatment of polarization and small backbone fluctuations, the effects which would lower the dielectric constant significantly if treated explicitly[26-31]. The overall binding energy, $-\Delta G_3$, was thus calculated by $-\Delta G_3 = \Delta G_1 + \Delta G_2 - \Delta G_4$. The components were calculated as following; ΔG_2 and ΔG_4 were computed using Adaptive Possion-Boltzmann Solver (APBS) to solve the linearized Poisson-Boltzmann equation[32,33]. ΔG_1 was calculated using CHARMM (v46b1) software[34].

Supplementary tables

Supplementary Table 1. *Mj*TyrRS gene library used in this study to select *Mj*TyrRS towards HYs

AA Position	*Mj*TyrRS	Library	Degenerate Codons
32	Y	All AAs except C, F, W, Y	VNK
65	L	All AAs	NNS
67	A	A, G, L, V	GBC / CTC
70	H	A, G, L, V	GBC / CTC
108	F	All AAs	NNS
109	Q	All AAs except W	NDT / VHG
158	D	A, G, L, V	GBC / CTC
162	L	All AAs except W	NDT / VHG

Supplementary Table 2. Sequences of aaRS mutant variants with specificity towards Br_2Y and Cl_2Y. BX (X: number) represents the clones from the Figure S2a plate containing Br_2Y while CX (X: number) shows the clones from the Figure S2a plate containing Cl_2Y. BXRS (X: number) and CXRS (X: number) represent the corresponding selected aaRS towards Br_2Y and Cl_2Y.

**Clone**	**Synthetase**	**Position**							
		**Y32**	**L65**	**A67**	**H70**	**F108**	**Q109**	**D158**	**L162**
WT	*Mj*TyrRS	Y	L	A	H	F	Q	D	L
B4	B4RS	V	E	A	G	Q	Q	A	C
B12	B12RS	L	E	A	G	T	Q	A	P
B37	B37RS	G	E	A	G	N	Q	A	Q
B39	B39RS	H	E	A	G	L	Q	A	A
B48	B48RS	G	E	A	G	Q	Q	A	N
C5	C5RS	G	E	A	G	N	S	A	E
C45	C45RS	A	E	A	G	E	Q	A	C
C61	C61RS	V	E	A	G	A	Q	A	Q
C64	C64RS	V	E	A	G	Q	Q	A	C

Supplementary Table 3. Expected and observed molecular weights of FtsZ-YFP-mts variants measured with ESI-MS*.

FtsZ-YFP-mts variants	Expected mass (Da)	Observed mass (Da)
Y (black line)	68061.29	68060.71
ClY (blue line)	68095.73	68094.02
Cl_2Y (yellow line)	68130.29	68130.04
BrY (green line)	68140.18	68139.11
Br_2Y (purple line)	68219.08	68219.41
IY (grey line)	68187.19	68186.04
I_2Y (red line)	68313.08	68312.59

*The accuracy of ESI-MS for intact protein molecules when using modern quadrupole time-of-flight (QTOF) MS has been documented to be 20 p.p.m when compared with the theoretical value[35].

Supplementary Table 4. Average ring diameter and treadmilling dynamics of Wild type FtsZ-YFP-mts and halogenated FtsZ-YFP-mts. n: ring numbers used for determining velocities.

**Protein**	**Average ring diameter (µm)**	**Treadmilling velocity (nm/s)**
Wild type FtsZ-YFP-mts	0.80±0.18, n=149	21.63±5.11, n=115
FtsZ(Y222ClY)-YFP-mts	0.62±0.15, n=150	15.10±4.78, n=93
FtsZ(Y222Cl_2Y)-YFP-mts	No ring	No ring
FtsZ(Y222BrY)-YFP-mts	0.89±0.19, n=150	15.61±6.15, n=101
FtsZ(Y222 Br_2Y)-YFP-mts	0.92±0.18, n=150	14.52±4.49, n=93
FtsZ(Y222 IY)-YFP-mts	No ring	No ring
FtsZ(Y222 I_2Y)-YFP-mts	1.03±0.21, n=149	11.36±4.19, n=104

Supplementary Table 5. Atomic partial charges calculated for the tyrosine mutants shown in Fig. 4b using Gaussian 16 and RESP tools integrated in Amber software. The backbone CA was replaced by a methyl group; one hydrogen was removed from the methyl group to merge with the peptide backbone and the charge was redistributed to the CB and HB atoms in order to give an overall neutral charge. Backbone charges were taken directly from the tyrosine parameters from Amber force field. Basis sets used for the calculation of the electrostatic potential (ESP) are listed in the table.

	Cl_2Y, deprot	
Basis Set	6-31G*	
Charges	CB	-0.088522
	CG	-0.017128
	CD1	-0.258483
	CD2	-0.258483
	CE1	-0.047197
	CE2	-0.047197
	CZ	0.497556
	OH	-0.777270
	Cl1	-0.157343
	Cl2	-0.157343
	HB2	0.028949
	HB3	0.028949
	HD1	0.140856
	HD2	0.140856
	-	
	N	-0.4157
	H	0.2719
	CA	-0.0014
	HA	0.0876
	C	0.5973

O	-0.5679

Supplementary Table 6. XYZ coordinates of 2,6-dichloro-4-methylphenolate after geometry optimization

CB	1	-0.24215	3.351338	-0.004059
CG	2	-0.13521	1.833026	-0.006476
CD1	3	-1.318698	1.077129	0.003353
CD2	4	1.142828	1.24217	0.000326
CE1	5	-1.219918	-0.339489	-0.003036
CE2	6	1.246207	-0.151132	0.010944
CZ	7	0.071039	-0.895503	0.011775
OM	8	0.249437	-2.204104	-0.023587
Cl1	9	-2.468533	-1.161441	0.004776
Cl2	10	2.575478	-0.827044	0.003721
HB1	11	0.43412	3.817144	-0.736895
HB2	12	0.005252	3.802511	0.972084
HD3	13	-1.259964	3.678975	-0.252759
HD1	14	-2.291942	1.564658	-0.003648
HD2	15	2.03438	1.868555	-0.011499

Supplementary Table 7 Experimental distribution coefficients (octan-1-ol/buffer) for amino acid derivatives.

structure	log*D*			
	pH 6	pH 7	pH 8	pH 9
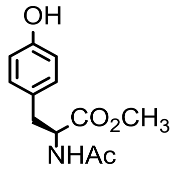	+0.28±0.04	+0.43±0.08	+0.29±0.03	+0.32±0.03
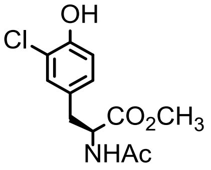	+0.94±0.04	+1.00±0.06	+0.76±0.07	+0.34±0.04
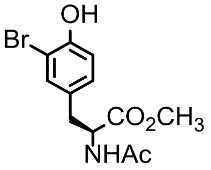	+1.10±0.05	+1.17±0.04	+0.96±0.07	+0.49±0.06
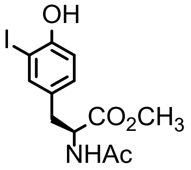	+1.49±0.06	+1.58±0.04	+1.29±0.02	+0.79±0.03
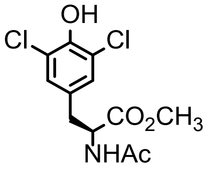	+1.50±0.06	+1.16±0.04	+0.15±0.02	−0.72±0.04
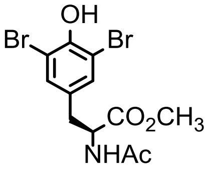	+1.72±0.05	+1.33±0.08	+0.34±0.03	−0.49±0.08
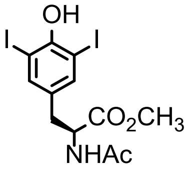	+2.18±0.05	+1.95±0.05	+0.85±0.04	0.00±0.04

Supplementary Table 8. List of plasmids used in this study

Plasmid	Replication Origin	Resistance	Description
pBU18'1GK_ *Mj*TyrRS_ Library	pUC	AmpR	*Mj*TyrRS Library (Supplementary Figure 1-2)
pPAB26_cat (Q98TAG, D181TAG) *Mj*tRNATyrCUA-his-SUMO-sfGFPR2TAG-strep	p15A	KanR	One-round Selection plasmid (Fig.S1, Fig.S2); SUMO-sfGFP production (Supplementary Figure 3)
pBU18'1GK_ B4RS(B4RS)	pUC	AmpR	tRNA Synthetase (Supplementary Figure 1-2); B48RS and C64RS were used for SUMO-sfGFP production (Supplementary Figure 3)
pBU18'1GK_ B12RS(B12RS)	pUC	AmpR	
pBU18'1GK_ B37RS(B372RS)	pUC	AmpR	
pBU18'1GK_ B39RS(B39RS)	pUC	AmpR	
pBU18'1GK_ C5RS(C12RS)	pUC	AmpR	
pBU18'1GK_ C45RS(C45RS)	pUC	AmpR	
pBU18'1GK_ C61RS(C61RS)	pUC	AmpR	
pBU18'1GK_ B48RS(B48RS)	pUC	AmpR	
pBU18'1GK_ C64RS(C64RS)	pUC	AmpR	
pULTRA_B48RS_tRNATyrCUA	CloDF13	SpecR	Halogenated FtsZ Protein expression (Fig.1.d.e.f, Fig.2, Fig.3 Supplementary Figure 4)
pULTRA_C64RS_tRNATyrCUA	CloDF13	SpecR	
pET28a_ FtsZ (Y222TAG)-YFP-mts	ColE1	KanR	
pET11b_wt_FtsZ-YFP-mts*	ColE1	AmpR	Wild type FtsZ protein expression (Fig.1.d.e.f, Fig.2, Fig.3, Supplementary Figure 4)

AmpR: Ampicillin resistance; KanR: Kanamycin resistance; SpecR: Spectinomycin resistance. *Ramirez-Diaz DA, García-Soriano DA, Raso A, Mücksch J, Feingold M, Rivas G, Schwille P: **Treadmilling analysis reveals new insights into dynamic FtsZ ring architecture**. *PLoS biology* 2018, **16**:e2004845.

Gene constructs

Nucleotide sequence of *Mj*TyrRS

Mutated amino acids are highlighted with red color

ATGGACGAATTTGAAATGATAAAGAGAAACACATCTGAAATTATCAGCGAGGAAGAGT

TAAGAGAGGTTTTAAAAAAAGATGAAAAATCTGCTTACATAGGTTTTGAACCAAGTGG

TAAAATACATTTAGGGCATTATCTCCAAATAAAAAAGATGATTGATTTACAAAATGCTGG

ATTTGATATAATTATATTGTTGGCTGATTTACATGCCTATTTAAACCAGAAAGGAGAGTT

GGATGAGATTAGAAAAATAGGAGATTATAACAAAAAAGTTTTTGAAGCAATGGGGTTA

AAGGCAAAATATGTTTATGGAAGTGAATTCCAGCTTGATAAGGATTATACACTGAATGT

CTATAGATTGGCTTTAAAAACTACCTTAAAAAGAGCAAGAAGGAGTATGGAACTTATAG

CAAGAGAGGATGAAAATCCAAAGGTTGCTGAAGTTATCTATCCAATAATGCAGGTTAAT

GATTCTCATTATTTAGGCGTTGATGTTGCAGTTGGAGGGATGGAGCAGAGAAAAATACA

CATGTTAGCAAGGGAGCTTTTACCAAAAAAGGTTGTTTGTATTCACAACCCTGTCTTAA

CGGGTTTGGATGGAGAAGGAAAGATGAGTTCTTCAAAAGGGAATTTTATAGCTGTTGA

TGACTCTCCAGAAGAGATTAGGGCTAAGATAAAGAAAGCATACTGCCCAGCTGGAGTT

GTTGAAGGAAATCCAATAATGGAGATAGCTAAATACTTCCTTGAATATCCTTTAACCATA

AAAAGGCCAGAAAAATTTGGTGGAGATTTGACAGTTAATAGCTATGAGGAGTTAGAGA

GTTTATTTAAAAATAAGGAATTGCATCCAATGtATTTAAAAAATGCTGTAGCTGAAGAAC

TTATAAAGATTTTAGAGCCAATTAGAAAGAGATTATAA

Nucleotide sequence of Chloramphenicol Acetyltransferase (CAT(Q98TAG, D181TAG))

Nucleotide sequence CAT(Q98TAG, D181TAG) with 2 amber stop - codons highlighted with red colour at position 98 and 181.

GATATCTGGCGAAAATGAGACGTTGATCGGCACGTAAGAGGTTCCAACTTTCAC

CATAATGAAATAAGATCACTACCGGGCGTATTTTTTGAGTTATCGAGATTTTCAGGAG

CTAAGGAAGCTAAAATGGAGAAAAAAATCACTGGATATACCACCGTTGATATATCCC

AATGGCATCGTAAAGAACATTTTGAGGCATTTCAGTCAGTTGCTCAATGTACCTATAA

CCAGACCGTTCAGCTGGATATTACGGCCTTTTTAAAGACCGTAAAGAAAAATAAGCA

CAAGTTTTATCCGGCCTTTATTCACATTCTTGCCCGCCTGATGAATGCTCATCCGGAAT

TTCGTATGGCAATGAAAGACGGTGAGCTGGTGATATGGGATAGTGTTCACCCTTGTTA

CACCGTTTTCCATGAGTAGACTGAAACGTTTTCATCGCTCTGGAGTGAATACCACGA

CGATTTCCGGCAGTTTCTACACATATATTCGCAAGATGTGGCGTGTTACGGTGAAAAC

CTGGCCTATTTCCCTAAAGGGTTTATTGAGAATATGTTTTTCGTCTCAGCCAATCCCTG

GGTGAGTTTCACCAGTTTTGATTTAAACGTGGCCAATATGGACAACTTCTTCGCCCCC

GTTTTCACCATGGGCAAATATTATACGCAAGGCTAGAAGGTGCTGATGCCGCTGGC

GATTCAGGTTCATCATGCCGTCTGTGATGGCTTCCATGTCGGCAGAATGCTTAATGAA

TTACAACAGTACTGCGATGAGTGGCAGGGCGGGGCGTAA

Nucleotide sequence of SUMO-His-sfGFP(R2TAG)

In-frame TAG stop codon is highlighted in red.

ATGGGCAGCAGCCATCATCATCATCATCACGGTTCTGACTCCGAAGTCAATCAAGAAG

CTAAGCCAGAGGTCAAGCCAGAAGTCAAGCCTGAGACTCACATCAATTTAAAGGTGTC

CGATGGATCTTCAGAGATCTTCTTCAAGATCAAAAAGACCACTCCTTTAAGAAGGCTG

ATGGAAGCGTTCGCTAAAAGACAGGGTAAGGAAATGGACTCCTTAAGATTCTTGTACG

ACGGTATTAGAATCCAAGCTGATCAGACCCCTGAAGATTTGGACATGGAGGATAACGAT

ATTATTGAGGCTCACCGCGAACAGATTGGTGGCATGTAGAAAGGCGAAGAGCTGTTCA

CTGGTGTCGTCCCTATTCTGGTGGAACTGGATGGTGATGTCAACGGTCATAAGTTTTCC

GTGCGTGGCGAGGGTGAAGGTGACGCAACTAATGGTAAACTGACGCTGAAGTTCATCT

GTACTACTGGTAAACTGCCGGTACCTTGGCCGACTCTGGTAACGACGCTGACTTATGGT

GTTCAGTGCTTTGCTCGTTATCCGGACCATATGAAGCAGCATGACTTCTTCAAGTCCGC

CATGCCGGAAGGCTATGTGCAGGAACGCACGATTTCCTTTAAGGATGACGGCACGTAC

AAAACGCGTGCGGAAGTGAAATTTGAAGGCGATACCCTGGTAAACCGCATTGAGCTG

AAAGGCATTGACTTTAAAGAAGACGGCAATATCCTGGGCCATAAGCTGGAATACAATTT

TAACAGCCACAATGTTTACATCACCGCCGATAAACAAAAAAATGGCATTAAAGCGAAT

TTTAAAATTCGCCACAACGTGGAGGATGGCAGCGTGCAGCTGGCTGATCACTACCAGC

AAAACACTCCAATCGGTGATGGTCCTGTTCTGCTGCCAGACAATCACTATCTGAGCAC

GCAAAGCGTTCTGTCTAAAGATCCGAACGAGAAACGCGATCATATGGTTCTGCTGGAG

TTCGTAACCGCAGCGGGCATCACGCATGGTATGGATGAACTGTACAAAAGCGCTTGGA
GCCACCCGCAGTTCGAAAAATAA

Nucleotide sequence of FtsZ(Y222TAG)-YFP-mts

Y222 is highlighted in red.

ATGTTTGAACCAATGGAACTTACCAATGACGCGGTGATTAAAGTCATCGGC
GTCGGCGGCGGCGGCGGTAATGCTGTTGAACACATGGTGCGCGAGCGCAT
TGAAGGTGTTGAATTCTTCGCGGTAAATACCGATGCACAAGCGCTGCGTAA
AACAGCGGTTGGACAGACGATTCAAATCGGTAGCGGTATCACCAAAGGAC
TGGGCGCTGGCGCTAATCCAGAAGTTGGCCGCAATGCGGCTGATGAGGATC
GCGATGCATTGCGTGCGGCGCTGGAAGGTGCAGACATGGTCTTTATTGCTG
CGGGTATGGGTGGTGGTACCGGTACAGGTGCAGCACCAGTCGTCGCTGAA
GTGGCAAAAGATTTGGGTATCCTGACCGTTGCTGTCGTCACTAAGCCTTTC
AACTTTGAAGGCAAGAAGCGTATGGCATTCGCGGAGCAGGGGATCACTGA
ACTGTCCAAGCATGTGGACTCTCTGATCACTATCCCGAACGACAAACTGCT
GAAAGTTCTGGGCCGCGGTATCTCCCTGCTGGATGCGTTTGGCGCAGCGAA
CGATGTACTGAAAGGCGCTGTGCAAGGTATCGCTGAACTGATTACTCGTCC
GGGTTTGATGAACGTGGACTTTGCAGACGTACGCACCGTAATGTCTGAGAT
GGGCTACGCAATGATGGGTTCTGGCGTGGCGAGCGGTGAAGACCGTGCGG
AAGAAGCTGCTGAAATGGCTATCTCTTCTCCGCTGCTGGAAGATATCGACC
TGTCTGGCGCGCGCGGCGTGCTGGTTAACATCACGGCGGGCTTCGACCTGC
GTCTGGATGAGTTCGAAACGGTAGGTAACACCATCCGTGCATTTGCTTCCG
ACAACGCGACTGTGGTTATCGGTACTTCTCTTGACCCGGATATGAATGACG
AGCTGCGCGTAACCGTTGTTGCGACAGGTATCGGCATGGACAAACGTCCTG
AAATCACTCTGGTGACCAATAAGCAGGTTCAGCAGCCAGTGATGGATCGCT
ACCAGCAGCATGGGATGGCTCCGCTGACCCAGGAGCAGAAGCCGGTTGCT
AAAGTCGTGAATGACAATGCGCCGCAAACTGCGCCCCCTCGACCTGCAGG
CGGCCGCATGGTGAGCAAGGGCGAGGAGCTGTTCACCGGGGTGGTGCCCA
TCCTGGTCGAGCTGGACGGCGACGTAAACGGCCACAAGTTCAGCGTGTCC

GGCGAGGGCGAGGGCGATGCCACCTACGGCAAGCTGACCCTGAAGCTGAT
CTGCACCACCGGCAAGCTGCCCGTGCCCTGGCCCACCCTCGTGACCACCC
TGGGCTACGGCCTGCAGTGCTTCGCCCGCTACCCCGACCACATGAAGCAGC
ACGACTTCTTCAAGTCCGCCATGCCCGAAGGCTACGTCCAGGAGCGCACC
ATCTTCTTCAAGGACGACGGCAACTACAAGACCCGCGCCGAGGTGAAGTT
CGAGGGCGACACCCTGGTGAACCGCATCGAGCTGAAGGGCATCGACTTCA
AGGAGGACGGCAACATCCTGGGGCACAAGCTGGAGTACAACTACAACAGC
CACAACGTCTATATCACCGCCGACAAGCAGAAGAACGGCATCAAGGCCAA
CTTCAAGATCCGCCACAACATCGAGGACGGCGGCGTGCAGCTCGCCGACC
ACTACCAGCAGAACACCCCCATCGGCGACGGCCCCGTGCTGCTGCCCGAC
AACCACTACCTGAGCTACCAGTCCGCCCTGAGCAAAGACCCCAACGAGAA
GCGCGATCACATGGTCCTGCTGGAGTTCGTGACCGCCGCCGGGATCACTCT
CGGCATGGACGAGCTGTACAAGGGCGGCCGCTTCATTGAAGAAGAGAAGA
AAGGCTTCCTCAAACGCTTGTTCGGAGGA

Supplementary References

1 Kubyshkin, V. Experimental lipophilicity scale for coded and noncoded amino acid residues. *Org. Biomol. Chem.* **19**, 7031-7040 (2021).

2 Yang, J. & Zhang, Y. I-TASSER server: new development for protein structure and function predictions. *Nucleic Acids Res.* **43**, W174-W181 (2015).

3 Oliva, M. A., Cordell, S. C. & Löwe, J. Structural insights into FtsZ protofilament formation. *Nat. Struct. Mol. Biol.* **11**, 1243-1250 (2004).

4 Šali, A. & Blundell, T. L. Comparative protein modelling by satisfaction of spatial restraints. *J. Mol. Biol.* **234**, 779-815 (1993).

5 Meyer, T. & Knapp, E.-W. p K a Values in Proteins Determined by Electrostatics Applied to Molecular Dynamics Trajectories. *J. Chem. Theory Comput.* **11**, 2827-2840 (2015).

6 Meyer, T., Kieseritzky, G. & Knapp, E. W. Electrostatic pKa computations in proteins: Role of internal cavities. *Proteins: Struct. Funct. Genet.* **79**, 3320-3332 (2011).

7 Case, D. A. *et al.* The Amber biomolecular simulation programs. *J. Comput. Chem.* **26**, 1668-1688 (2005).

8 Thomas, S. R., McTamney, P. M., Adler, J. M., LaRonde-LeBlanc, N. & Rokita, S. E. Crystal structure of iodotyrosine deiodinase, a novel flavoprotein responsible for iodide salvage in thyroid glands. *J. Biol. Chem.* **284**, 19659-19667 (2009).

9 Liu, X. *et al.* Significant expansion of fluorescent protein sensing ability through the genetic incorporation of superior photo-induced electron-transfer quenchers. *J. Am. Chem. Soc.* **136**, 13094-13097 (2014).

10 Frisch, M. *et al.* Gaussian 16, Revision C. 01. Gaussian, Inc., Wallingford CT. 2016.

11 Rassolov, V. A., Ratner, M. A., Pople, J. A., Redfern, P. C. & Curtiss, L. A. 6-31G* basis set for third-row atoms. *J. Comput. Chem.* **22**, 976-984 (2001).

12 Dobbs, K. & Hehre, W. Molecular orbital theory of the properties of inorganic and organometallic compounds. 6. Extended basis sets for second-row transition metals. *J. Comput. Chem.* **8**, 880-893 (1987).

13 Wang, J., Wang, W., Kollman, P. A. & Case, D. A. Automatic atom type and bond type perception in molecular mechanical calculations. *J. Mol. Graph. Model.* **25**, 247-260 (2006).

14 Eargle, J., Wright, D. & Luthey-Schulten, Z. Multiple Alignment of protein structures and sequences for VMD. *Bioinformatics* **22**, 504-506 (2006).

15 Maier, J. A. *et al.* ff14SB: improving the accuracy of protein side chain and backbone parameters from ff99SB. *J. Chem. Theory Comput.* **11**, 3696-3713 (2015).

16 Meagher, K. L., Redman, L. T. & Carlson, H. A. Development of polyphosphate parameters for use with the AMBER force field. *J. Comput. Chem.* **24**, 1016-1025 (2003).

17 Jorgensen, W. L., Chandrasekhar, J., Madura, J. D., Impey, R. W. & Klein, M. L. Comparison of simple potential functions for simulating liquid water. *J. Chem. Phys.* **79**, 926-935 (1983).

18 Feller, S. E., Zhang, Y., Pastor, R. W. & Brooks, B. R. Constant pressure molecular dynamics simulation: The Langevin piston method. *J. Chem. Phys.***103**, 4613-4621 (1995).

19 Ryckaert, J.-P., Ciccotti, G. & Berendsen, H. J. Numerical integration of the cartesian equations of motion of a system with constraints: molecular dynamics of n-alkanes. *J. Comput. Phys.* **23**, 327-341 (1977).

20 Darden, T., York, D. & Pedersen, L. Particle mesh Ewald: An N· log (N) method for Ewald sums in large systems. *J. Chem. Phys.* **98**, 10089-10092 (1993).

21 Budisa, N. *et al.* Residue-specific bioincorporation of non-natural, biologically active amino acids into proteins as possible drug carriers: structure and stability of the per-thiaproline mutant of annexin V. *Proc. Natl. Acad. Sci.* **95**, 455-459 (1998).

22 Crooks, G. E., Hon, G., Chandonia, J.-M. & Brenner, S. E. WebLogo: a sequence logo generator. *Genome Res.* **14**, 1188-1190 (2004).

23 Renner, L. D. & Weibel, D. B. MinD and MinE interact with anionic phospholipids and regulate division plane formation in Escherichia coli. *J. Biol. Chem.* **287**, 38835-38844 (2012).

24 Caprioli, R. M., Malorni, A. & Sindona, G. *Mass spectrometry in biomolecular sciences*. Vol. 475 (Springer Science & Business Media, 2012).

25 Hvattum, E. & Priebe, H. Deiodination of iodinated aromatic compounds with electrospray ionization mass spectrometry. *Rapid Communications in Mass Spectrometry* **27**, 2504-2514 (2013).

26 Rabenstein, B., Ullmann, G. M. & Knapp, E.-W. Energetics of electron-transfer and protonation reactions of the quinones in the photosynthetic reaction center of Rhodopseudomonas viridis. *Biochemistry* **37**, 2488-2495 (1998).

27 Rosen, D. Dielectric properties of protein powders with adsorbed water. *J. Chem. Soc. Faraday Trans.* **59**, 2178-2191 (1963).

28 Gilson, M. K. & Honig, B. Calculation of the total electrostatic energy of a macromolecular system: solvation energies, binding energies, and conformational analysis. *Proteins: Struct. Funct. Genet.* **4**, 7-18 (1988).

29 Gilson, M. K. & Honig, B. H. The dielectric constant of a folded protein. *Biopolymers: Original Research on Biomolecules* **25**, 2097-2119 (1986).

30 Warshel, A. & Papazyan, A. Electrostatic effects in macromolecules: fundamental concepts and practical modeling. *Curr. Opin. Struct. Biol.* **8**, 211-217 (1998).

31 Schutz, C. N. & Warshel, A. What are the dielectric “constants” of proteins and how to validate electrostatic models? *Proteins: Struct. Funct. Genet.* **44**, 400-417 (2001).

32 Baker, N. A., Sept, D., Joseph, S., Holst, M. J. & McCammon, J. A. Electrostatics of nanosystems: application to microtubules and the ribosome. *Proc. Natl. Acad. Sci.* **98**, 10037-10041 (2001).

33 Holst, M. & Saied, F. Multigrid solution of the Poisson—Boltzmann equation. *J. Comput. Chem.* **14**, 105-113 (1993).

34 Brooks, B. R. *et al.* CHARMM: a program for macromolecular energy, minimization, and dynamics calculations. *J. Comput. Chem.* **4**, 187-217 (1983).

35 Donnelly, D. P. *et al.* Best practices and benchmarks for intact protein analysis for top-down mass spectrometry. *Nat. Methods.* **16**, 587-594 (2019).
