# Supplementary material for: Halogenation of tyrosine perturbs large-scale protein self-organization
Source: Nat Commun. 2022 Aug 17;13:4843. doi: 10.1038/s41467-022-32535-2 (PMC9385671; doi:10.1038/s41467-022-32535-2)
Supplement: Supplementary file 3 — Description of additional supplementary files [file 41467_2022_32535_MOESM3_ESM.pdf]

Description Of Additional Supplementary Files

Supplementary Movie 1 : Selforganization of wild type FtsZ-YFPmts and halogenated FtsZ(Y222X)- YFP-mts (X = ClY, BrY, Br2Y, I2Y, Cl2Y, and IY) on supported membrane (0.5 µM proteins, 4 mM GTP and 1 mM Mg2+).

Supplementary Movie 2 : Selforganization dynamics of wild type FtsZ-YFP-mts and FtsZ(Y222 Cl2Y)-YFP-mts at certain proportions on supported membrane (0.5 µM proteins, 4 mM GTP and 1 mM Mg2+)
