# Supplementary material for: Halogenation of tyrosine perturbs large-scale protein self-organization
Source: Nat Commun. 2022 Aug 17;13:4843. doi: 10.1038/s41467-022-32535-2 (PMC9385671; doi:10.1038/s41467-022-32535-2)
Supplement: Supplementary file 6 — Reporting Summary [file 41467_2022_32535_MOESM6_ESM.pdf]

nature portfolio

Corresponding author(s): Nediljko Budisa Petra Schwille Andrea Mroginski and Haiyang Jia

Last updated by author(s): Jul 25, 2022

Reporting Summary

Nature Portfolio wishes to improve the reproducibility of the work that we publish. This form provides structure for consistency and transparency in reporting. For further information on Nature Portfolio policies, see our Editorial Policies and the Editorial Policy Checklist.

Statistics

For all statistical analyses, confirm that the following items are present in the figure legend, table legend, main text, or Methods section.

n/a	Confirmed	
☐	☒	The exact sample size (*n*) for each experimental group/condition, given as a discrete number and unit of measurement
☐	☒	A statement on whether measurements were taken from distinct samples or whether the same sample was measured repeatedly
☐	☒	The statistical test(s) used AND whether they are one- or two-sided<br>*Only common tests should be described solely by name; describe more complex techniques in the Methods section.*
☒	☐	A description of all covariates tested
☒	☐	A description of any assumptions or corrections, such as tests of normality and adjustment for multiple comparisons
☐	☒	A full description of the statistical parameters including central tendency (e.g. means) or other basic estimates (e.g. regression coefficient) AND variation (e.g. standard deviation) or associated estimates of uncertainty (e.g. confidence intervals)
☐	☒	For null hypothesis testing, the test statistic (e.g. F, t, r) with confidence intervals, effect sizes, degrees of freedom and P value noted<br>*Give P values as exact values whenever suitable.*
☒	☐	For Bayesian analysis, information on the choice of priors and Markov chain Monte Carlo settings
☒	☐	For hierarchical and complex designs, identification of the appropriate level for tests and full reporting of outcomes
☒	☐	Estimates of effect sizes (e.g. Cohen's *d*, Pearson's *r*), indicating how they were calculated

Our web collection on statistics for biologists contains articles on many of the points above.

Software and code

Policy information about availability of computer code

Data collection	softWoRx Software (GE Healthcare Life Sciences, Germany) and Zeiss Zen software (Zen 2.1 black) were used to acquire images. VMD (v1.9.3) and Pymol(4.60) were used for visualisation of structures. Modeller and I-Tasser were used for homology modeling. Amber software was used for molecular dynamics simulations. Gaussian 16 was used for computation of partial charges of non-standard residues. CHARMM (v46b1) and APBS were used for computation of binding energies. Karlsberg2+ was used for pKa computations and is available upon request from authors (Prof. Ernst-Walter Knapp, knapp@chemie.fu-berlin.de). Topspin 3.6 was used for collecting the NMR data. MassHunter Workstation Software - LC/MS Data Acquisition (B06.01) was used for mass data collection.
Data analysis	Zeiss Zen software (Zen 2.1 black) , softWoRx Software (GE Healthcare Life Sciences, Germany) , imageJ 1.53f51(Fiji), , OriginPro software (v_2017 & v_2019) , Matlab R2016a, Pymol(4.60), VMD (v1.9.3), Python3 (MatPlotLib, Numpy).Spectra deconvolution was performed with Agilent MassHunter Qualitative Analysis software (v. B.06.00, Bioconfirm Intact mass module). Topspin 4.0 was used for analysis the NMR data. Code of ring velocity analysis is from the published approach(PLoS biology, 2018, 16(5): e2004845) and is available upon request from author (Prof. Petra Schwille, schwille@biochem.mpg.de).

For manuscripts utilizing custom algorithms or software that are central to the research but not yet described in published literature, software must be made available to editors and reviewers. We strongly encourage code deposition in a community repository (e.g. GitHub). See the Nature Portfolio guidelines for submitting code & software for further information.

Data

Policy information about availability of data

All manuscripts must include a data availability statement. This statement should provide the following information, where applicable:

- Accession codes, unique identifiers, or web links for publicly available datasets
- A description of any restrictions on data availability
- For clinical datasets or third party data, please ensure that the statement adheres to our policy

All data used in this paper are available at Figshare through the identifier https://doi.org/10.6084/m9.figshare.20368626.v1 or from the corresponding authors upon request. Source data are provided with this paper. All the data of protein structures are provided with this paper (structures.zip).

Field-specific reporting

Please select the one below that is the best fit for your research. If you are not sure, read the appropriate sections before making your selection.

☒ Life sciences ☐ Behavioural & social sciences ☐ Ecological, evolutionary & environmental sciences

For a reference copy of the document with all sections, see nature.com/documents/nr-reporting-summary-flat.pdf

Life sciences study design

All studies must disclose on these points even when the disclosure is negative.

Sample size	Samples sizes were limited by practicality and throughput. Unless otherwise mentioned, all measurements were performed for at least three independent experiments.
Data exclusions	Images were only excluded from analysis when the quality of the supported lipid bilayer was very low.
Replication	The numbers of experimental repeats are indicated in the respective figure legends. All observations were replicated successfully.
Randomization	For one-round positive selection of MjTyrRS variants, positive E. coli clones were randomly picked. There were no bias from the researchers, since all the positive clones looked the same. In the in vitro research, samples were mixed from scratch of protein components. Samples were not randomly assigned by chance to an experimental group and all data were considered equally. Therefore, randomization is not applicable for the in vitro research.
Blinding	For the in vitro research, blinding was not applicable, since the data collections and analysis are automatic, and all analysis was intrinsically without user-bias. Regarding the selection experiments, all the positive clones were similar in shape and the difference of fluorescence intensities were not distinguished for eyes , therefore blinding is not necessary when randomly picking clones.

Reporting for specific materials, systems and methods

We require information from authors about some types of materials, experimental systems and methods used in many studies. Here, indicate whether each material, system or method listed is relevant to your study. If you are not sure if a list item applies to your research, read the appropriate section before selecting a response.

Materials & experimental systems

n/a	Involved in the study	
☒	☐	Antibodies
☒	☐	Eukaryotic cell lines
☒	☐	Palaeontology and archaeology
☒	☐	Animals and other organisms
☒	☐	Human research participants
☒	☐	Clinical data
☒	☐	Dual use research of concern

Methods

n/a	Involved in the study	
☒	☐	ChIP-seq
☒	☐	Flow cytometry
☒	☐	MRI-based neuroimaging
